# Epidemiology of Multi-Drug Resistant Organisms in a Teaching Hospital in Oman: A One-Year Hospital-Based Study

**DOI:** 10.1155/2014/157102

**Published:** 2014-01-14

**Authors:** Abdullah Balkhair, Yahya M. Al-Farsi, Zakariya Al-Muharrmi, Raiya Al-Rashdi, Mansoor Al-Jabri, Fatma Neilson, Sara S. Al-Adawi, Marah El-Beeli, Samir Al-Adawi

**Affiliations:** ^1^Department of Infection Control, Sultan Qaboos University Hospital, Sultan Qaboos University, Muscat, Oman; ^2^Department of Family Medicine and Public Health, College of Medicine and Health Sciences, Sultan Qaboos University, P.O. Box 35, P.C. 123 Al-Khoud, Muscat, Oman; ^3^Oman Medical College, Sohar Campus, Al Tareef, Sohar, Oman; ^4^Department of Behavioral Medicine, College of Medicine and Health Sciences, Sultan Qaboos University, Muscat, Oman

## Abstract

*Background*. Antimicrobial resistance is increasingly recognized as a global challenge. A few studies have emerged on epidemiology of multidrug resistant organisms in tertiary care settings in the Arabian Gulf. *Aim*. To describe the epidemiology of multi-drug resistant organisms (MDRO) at Sultan Qaboos University Hospital, a tertiary hospital in Oman. *Methods*. A retrospective review of MDRO records has been conducted throughout the period from January 2012 till December 2012. Organisms were identified and tested by an automated identification and susceptibility system, and the antibiotic susceptibility testing was confirmed by the disk diffusion method. *Results*. Out of the total of 29,245 admissions, there have been 315 patients registered as MDRO patients giving an overall prevalence rate of 10.8 (95% CI 9.3, 12.4) MDRO cases per 1000 admissions. In addition, the prevalence rate of MDRO isolates was 11.2 (95% CI 9.7, 12.9) per 1000 admissions. Overall, increasing trends in prevalence rates of MDRO patients and MDRO isolates were observed throughout the study period. *Conclusion*. Antimicrobial resistance is an emerging challenge in Oman. Continuous monitoring of antimicrobial susceptibility and strict adherence to infection prevention guidelines are essential to prevent proliferation of MDRO. Along such quest, stringent antibiotic prescription guidelines are needed in the country.

## 1. Introduction

Data emerging from different parts of the world have suggested that strains of highly multidrug resistant organisms (MDRO) have quadrupled in the past decade [1, 2]. MDRO are increasingly being recognized to be impervious to available antibiotics. When MDRO afflict a vulnerable individual, the risk of morbidity and mortality is heightened. This has other insidious negative repercussions including lengthening of hospital stay and increase in social and medical costs due to the emergence of MDRO [3, 4]. Prior to the turn of the century, among the healthcare planners, there was enthusiasm that there will be “Health for all by the year 2000” [5]. In retrospect, this was a far-fetched aspiration. Barely two decades into a new century, there were proliferations of infectious diseases which render the standard of public health in many parts of the world equivalent to preantibiotic era [6].

There are likely to be myriads of factors that contribute to the rising tide of nosocomial infections. Firstly, the many attempts to find alternative means including bacteriophage [7] and immune-enhancing strategies [8] have not been found to be a practical adjunct antibiotic. Secondly, the development of “new generation of antibiotics” has been hampered with existing regulations that are not amenable for the development of new antibiotics such as the lack of scientific strategies to develop new class of antibiotics [9–11]. For these reasons, there has been a long lull on the development of new antibiotics. Some critics have also argued that pharmaceutical companies have little incentive to invest in what could be an enormously costly venture to develop new antibiotics, while the profit margin of the newly developed antibiotics is lower compared to “lifestyle” medications [12]. Thirdly, there is strong indication that the global proliferation of strains of multidrug resistant bacteria may partly stem from the fact that there is pervasive overuse and misuse of antimicrobial agents [13]. There has been a universal call of judicious use of antimicrobial agents, but such message appears to be unheeded, as emergence of various strains of intractable and antibiotic resistant organism appears to be proliferating in different parts of the world [6]. Fourthly, present proliferation of different strains of multidrug resistant organisms is limited to not only overuse in humans, but also the significant contribution of antibiotic resistant organism in food supply due to overuse/abuse of antibiotics for treatment, prevention, and growth promotion in animals [14]. As yet, there are no feasible strategies to reduce antibiotic-resistant organism from entering the food chain [15].

In the age of globalization, advances in transportation and telecommunications infrastructure have not only increased cross-cultural and technological interchange and aided in the diffusion of organisms from one region to another, but also created fertile ground for internationalization of multidrug resistant bacteria. Oman, a country in the southern tip of the Arabian Peninsula, overlooking the continent of Asia and Africa, represent the place where globalization and Arabia intertwine. According to the 2010 census [16], its population is estimated to be 2,773,479. The country is classified by the World Bank as a “high-income economy” among the emerging economies [17]. Recent affluence due to exploitation of hydrocarbon has turned Oman as a hub for international shipment and trade. The country has attracted a number of foreign workers from different parts of the world, with all the consequences this may entail in terms of human, animal, and material exchanges as testified by infections that were linked to zoonotic events [18]. Some preliminary studies have alluded to the existence of MDRO. In the year 2005, Al-Muharrmi et al. [19] evaluated extended-spectrum *β*-lactamase (ESBL) isolates in a pediatric population in Oman. For over 12-month period of observation, the authors found that 13.3% of *E. coli* and 6.6% of *Klebsiella pneumoniae* isolated were ESBL producers. The study concluded that ESBL-producing organisms are becoming a major problem in Omani children. In reference to the pediatric population, Al-Muharrmi and colleagues [20] also investigated the *in vitro* activity of carbapenems, piperacillin-tazobactam, and ciprofloxacin alone or in combination with aminoglycosides against ESBL-producing strains isolated from clinical samples. It was found that ESBLs have high resistance profiles against piperacillin/tazobactam and ciprofloxacin. The study concluded that the ESBLs from Oman have a similar resistance pattern to those reported from the UK and USA. Other studies in the regions have also pointed out the emergence of multidrug resistant organisms [21–23]. More recently, Aly and Balkhy [24] have synthesized the prevailing situation in the region by reviewing published articles on nosocomial infections. These authors identified a total of 45 articles published between 1990 and 2011. On the whole, the review indicates emergence of MDRO. Accordingly, *Escherichia coli, Klebsiella pneumoniae, Pseudomonas aeruginosa*, methicillin-resistant *Staphylococcus aureus*, *Acinetobacter C. difficile*, and *Enterococcus* do exist in the region with variable rates of such strains of MDRO. Most of the studies have focused on one particular subspecialty of clinical departments. Studies are needed to garner comprehensive view, for example, of tertiary care hospitals where attendees of such hospitals are likely to have advanced pathology of the disease and to be highly immunocompromised and, therefore, more prone to succumb to vagaries of MDRO. Therefore, exploring epidemiology and types of multidrug resistant organisms in a tertiary care is imperative. Oman represents a fertile ground for such undertaking. To our knowledge, there is no previous comprehensive document of MDRO in Oman.

In order to consolidate the available information of MDRO in a tertiary care setting in Oman, this study aims to address the monthly cumulative frequencies and prevalence rates of hospital-acquired multidrug resistant organisms during the year of 2012. It also aims to describe the distribution of hospital-acquired MDRO by site of infection and types of bacteria.

## 2. Methods

A retrospective review was conducted to all reports of Gram-negative and Gram-positive isolates from all units at SQUH which is a 570-bed tertiary care teaching hospital in Muscat, Oman. The study has been conducted for a period from January 2012 to December 2012.

Gram-negative and Gram-positive organisms were identified and tested. Definition of MDRO was based on the recently proposed joint definition by the European Center for Disease Prevention and Control (ECDC) and the Centers for Disease Prevention and Control (CDC) [25].

Two prevalence rates were calculated: (1) prevalence rate of MDRO patients and (2) prevalence rate of MDRO isolates. Both prevalence rates were calculated by dividing the number of cases with index by number of monthly inpatient admissions for the whole year. The prevalence rates were reported per 1,000 admissions. The 95% confidence intervals (95% CI) of prevalence rates were calculated using the Poisson distribution method of binomial variables. The 95% CI were calculated using the GraphPad Prism 6.0 software.

The trend in the prevalence rates over a 12-month period was calculated and analyzed to identify a statistically significant increasing or decreasing trend using the Cochran-Armitage test for trend, and a cutoff value for statistical significance was taken at *P* value of 0.05. Statistical Package for Social Sciences (SPSS) (version 20.0, IBM) was used for all statistical analyses. Ethical approval for this study was obtained from the Institutional Review Board of Sultan Qaboos University, Medical Research Ethics Committee at College of Medicine and Health Sciences.

## 3. Results

Table 1 shows the cumulative frequencies and rates of hospital-acquired MDRO among patients and isolates at SQUH during the year of 2012. Overall and of the total of 29,245 admissions, there has been 315 patients who were registered as hosting MDRO, giving an overall prevalence rate of 10.8 (95% CI 9.3, 12.4) MDRO cases per 1000 admissions. In addition, there were 329 registered MDRO isolates giving a prevalence rate of 11.2 (95% CI 9.7, 12.9) MDRO isolates per 1000 admissions. The lowest prevalence rate was observed in June (4.0; 95% CI 2.0, 7.2), while the highest prevalence rate was observed in October (17.7; 95% CI 12.9, 23.9).

**Figure 1:**
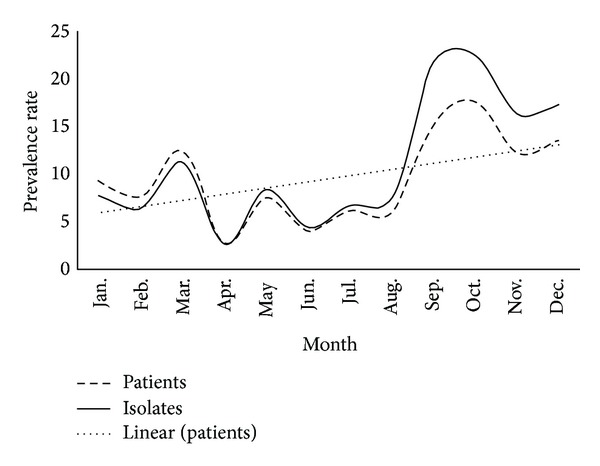
Prevalence rates of hospital-acquired multidrug resistant organisms (MDRO) at SQUH, Oman, 2012.

As shown in Figure 1, the prevalence rates of MDRO patients and isolates were relatively steady throughout the period from January to August 2012. In September, there was an abrupt increase in the prevalence rates which continuously elevated until the end of the year. Overall, the increasing trends for prevalence rates of MDRO patients and MDRO isolates were both found to be statistically significant (trend, *P* = 0.04, *P* = 0.03, resp.).

Table 2 depicts that bloodstream infection and pneumonia were the most frequently occurring infections caused by hospital-acquired MDRO (24.6% and 24.3%, resp.) followed by urinary tract infection (UTI) (18.8%). Surgical infections were encountered among 9.7% of all MDRO affected patients.

As illustrated in Table 3, the number of types of bacteria was nine, namely, *Acinetobacter baumannii*, methicillin-resistant *S. aureus* (MRSA), *Escherichia coli*, *Escherichia coli* (CRE), *Klebsiella pneuminae*, *Klebsiella pneumoniae* (ESBL), *Enterobacter*, *Pseudomonas*, *Enterococcus* (VRE), *Burkholderia,* and *Stenotrophomonas maltophilia*. *Acinetobacter baumannii* was the most prevalent MDRO among the isolates (32.4%). *E. coli* comes next with a percentage of 18.4% followed by methicillin-resistant *S. aureus* (MRSA) (10.6%) and *Klebsiella pneumoniae* (ESBL) (10.3%). The frequencies of *Pseudomonas aeruginosa*, *Klebsiella pneumoniae* (CRE), and *Stenotrophomonas maltophilia* were nearly equal (8.1%, 7.8%, and 7.5%, resp.). The rest were encountered at a frequency of less than 2%.

## 4. Discussion

It has been estimated that nosocomial infections annually contract around two million in North America with a significant number of them requiring critical care. The colonization of MDRO tends to have an unexpectedly high rate of morbidity and mortality. The social, economic, and medical cost of nosocomial infections mount to a huge financial cost [26]. Such situation is not limited to North America; there are preliminary studies indicating that healthcare-associated infections constitute a global challenge in the GCC countries [27]. Previous surveys of Omani healthcare-associated infections were limited to specific clinical populations [19, 20]. To our knowledge, this study is the first of its kind that comprehensively explores monthly variations, prevalence rates, sites of infection, and type of MDRO in a tertiary care hospital.

As for the first hypothesis regarding monthly variations, there was an average of 2437 admissions with approximately 22.4 patients with MDRO and approximately 27 isolates, suggesting that some afflicted individuals have had more than one organism. There is a strong indication that climatic factors tend to influence the rate of nosocomial infections [28]. This study indicates that the nosocomial infections are invariably affected by the weather. In this study, healthcare-associated infections were shown to tend to dwindle in summer. In support of this, the summer season which normally peaks from April to September appears to be associated with fewer incidents of healthcare-associated infections. If this finding would withstand further scrutiny, countermeasures to mitigate healthcare-associated infections should consider factors such as monthly variation and the climatic conditions under which infections are more likely to occur.

The second related aim of this study is to gauge prevalence rates of hospital-acquired multidrug resistant organisms. Overall, the prevalence rate of MDRO patients was found to be 10.8 per 1000 admissions. There has been considerable variation in reported prevalence estimates of MDRO worldwide that ranged from 6 and 31 per 1000 admissions [29]. Despite that, all reports documented an increasing trend over the last few years. The prevalence of MDRO reported in this study was 10.8 per 1000 admissions, which falls within the international range. Estimation of prevalence is of great importance as it provides the health authorities with reliable data to plan adequate health care measures. This study indicates an increasing trend throughout 12 months of observation. In addition to climatic conditions, the increased prevalence of MDRO has been linked to antimicrobial use, inadequate antimicrobial therapy, and long antimicrobial exposures that may trigger emergence of resistance in a bacterium that was previously amenable to antibiotics. There is strong evidence suggesting that contaminated healthcare workers' hands or environmental surfaces may play a role in exacerbating MDRO [30]. Other factors associated with MDRO include gender, underlying comorbidity as well as those who are exposed to medical gadgets that cross different anatomical sites and breach the body's natural physical barriers, such as mechanical ventilations and catheters [31, 32].

The third aim of this study is to quantify the distribution of sites of infection at this particular tertiary care. The commonest site of MDRO infection was bloodstream and pneumonia. El-Saed et al. [33] reported that the most common sites in their study in Saudi Intensive Care Unit at a tertiary hospital for MDRO were respiratory and blood. Similar pattern was observed in other countries including Italy [34] and Finland [35]. However, other studies have pointed out different distributions for sites of MDRO infection. For example, Jaggi [36] traced bacterial cultures over a period of 45 months in a tertiary care hospital in Haryana, India. It was found that MDRO was isolated mostly from urine cultures which constituted 38% of the total bacterial cultures. Although the emergence of MDRO is a global phenomenon, it is likely to be across populations and geographical variations. Therefore, it was not surprising that, in Oman, the rate of MDRO appears to have monthly variations.

The final related aim of this study was to survey the commonest types of MDRO. Decades of research have identified various common multidrug resistant organisms including Vancomycin-resistant *Enterococci*, MRSA, *Klebsiella pneumonia*, carbapenemase producing Gram-negatives, *Enterococcus faecium*, *Staphylococcus aureus*, *Acinetobacter baumannii,* as well as those that fall under the broad category of multidrug resistant gram-negative rods. The most common MDRO were those related to *Enterobacter* species, *Acinetobacter baumannii*. The reported array of organisms in this study has similarities with other studies conducted in theregion. In an attempt to review the prevalence of antibiotic resistance in the GCC countries and the reasons behind this phenomenon, Aly and Balkhy [24] reported that the most prevalent microorganism was *E. coli* (44%) followed by *K. pneumoniae* (20%) and *P. aeruginosa* (18.7%). MRSA and *Acinetobacter *were found to be less frequently observed by nearly equal percentages (5.4% and 5%, resp.). In a previous study done on patients admitted to ICU in Riyadh Military Hospital in Saudi Arabia, similar isolates were obtained among which* Acinetobacter baumannii* was the most common MDRO (40.9%). In Saudi Arabia also, El Tahawy and Khalaf [37] evaluated 100 isolates and found that *P. aeruginosa*, *K. pneumoniae*, *E. coli*, and *Enterobacter* were the most commonly isolated, with imipenem, ciprofloxacin, and amikacin showing greatest efficacy. Another study carried out at a tertiary care hospital in Riyadh [38] over a one-year period showed the most frequent pathogens to be *P. aeruginosa, E. coli, S. aureus, K. pneumoniae*, and *S. marcescens*.

Our study is not without limitations. This single center study data may not reflect the MDRO prevalence trend in Oman as a whole. Therefore, a multisite study would be essential in order to lay groundwork for comparison between different healthcare settings. Another limitation is lack of certainty that all clinical specimens represented active infection. Not all of the isolates may represent active infections from the patients. In addition, we did not correlate the samples to admission data for each patient/clinical specimen, so we were unable to provide a more accurate description of community versus nosocomial onset of infection. Studies with more robust methodology would be needed in order to generalize the present findings.

In conclusion, this study demonstrated an increasing trend of MDRO in Oman, with an array of organisms that was similar to those reported in neighboring countries. These findings call for the prompt necessity in developing nationwide antibiotic policy and guidelines, which is essential nowadays due to the increasing resistance patterns. In addition, it calls for developing a local antibiogram database which will improve the knowledge of antimicrobial resistance patterns in Oman and will also help to improve treatment strategies based on unit-specific data.

## Figures and Tables

**Table 1:** Cumulative frequencies and rates of hospital-acquired multidrug resistant organisms (MDRO) among patients and isolates at SQUH, Oman, 2012.

				Rate per patient	Rate per isolate
Month	Total admissions	MDRO patients	MDRO isolates	*R* (95% CI)^a^	*R* (95% CI)^a^
January	2699	25	21	9.3 (4.8, 11.9)	7.8 (5.9, 11.9)
February	2506	19	16	7.6 (3.6, 10.4)	6.4 (4.4, 12.0)
March	2506	31	28	12.4 (7.6, 16.4)	11.2 (8.4, 17.6)
April	2561	9	7	2.7 (16.0, 28.1)	2.7 (1.2, 5.5)
May	2523	19	21	7.5 (4.4, 11.9)	8.3 (5.2, 12.7)
June	2488	10	11	4.0 (2.0, 7.2)	4.4 (2.0, 8.0)
July	2405	15	16	6.2 (3.3, 10.0)	6.7 (3.7, 10.8)
August	2150	13	16	6.0 (3.3, 10.2)	7.4 (4.2, 12.1)
September	2347	36	51	15.3 (10.7, 21.3)	21.7 (16.2, 28.2)
October	2255	40	51	17.7 (12.9, 23.9)	22.6 (16.9, 29.7)
November	2374	29	39	12.2 (8.0, 17.7)	16.4 (11.8, 22.3)
December	2431	33	42	13.6 (9.5, 18.9)	17.3 (12.3, 23.4)

Total	29245	315	329	10.8 (9.3, 12.4)	11.2 (9.7, 12.9)

^a^Rate calculated per 1000 admissions.

**Table 2:** Distribution of hospital-acquired multidrug resistant organisms by site of infection at tertiary care centre in Oman, 2012.

Site of infection	*N* (Total = 329)	%
Bloodstream infection	81	24.6
Pneumonia	80	24.3
Urinary tract infection	62	18.8
Surgical infection	32	9.7
Others	74	22.5

**Table 3:** Distribution of hospital-acquired multidrug resistant organisms (MDRO) by type of bacteria at a tertiary care hospital in Oman, 2012.

Type of bacteria	*N* (Total = 329)	%
*Acinetobacter baumannii *	107	32.4
*Escherichia coli* (ESBL)	60	18.4
Methicillin-resistant* Staphylococcus aureus *	35	10.6
*Klebsiella pneumoniae* (ESBL)	34	10.3
*Pseudomonas aeruginosa *	27	8.1
*Klebsiella pneumoniae* (CRE)	26	7.8
*Stenotrophomonas maltophilia *	25	7.5
*Escherichia coli* (CRE)	5	1.6
*Burkholderia cepacia *	5	1.6
*Enterobacter cloacae *	4	1.2
*Enterococcus faecalis/faecium* (VRE)	2	0.6

ESBL: extended spectrum *β*-lactamases; VRE: vancomycin-resistant *Enterococcus*; CRE: carbapenem-resistant *Enterobacteriaceae.*
